# Lectures on the Exploration and Treatment of Diseases of the Chest

**Published:** 1840-06-06

**Authors:** W. W. Gerhard


### MEDICAL EXAMINER.

DEVOTED TO MEDICINE, SURGERY, AND THE COLLATERAL SCIENCES.

No. 23.] PHILADELPHIA, SATURDAY, JUNE 6, 1840. [Vol. III.

### LECTURES ON THE EXPLORATION AND TREATMENT OF DISEASES OF THE CHEST.

BY W. W. GERHARD, M. D.

### Lecture IV. and V.—*Auscultation.*

We now come to the most important and extended means of physical exploration,—that is, auscultation, or the act of hearing and interpreting the sounds produced in the chest during the act of respiration, or of coughing or speaking, or caused by the action of the heart. Percussion teaches us merely the density of the tissue of the lungs; but auscultation goes much farther, and not only indicates the physical density of the tissue, but the functional play of the organs, and the obstructions which impede the passage of the air in the lungs, or of the blood in the heart. Hence the signs of auscultation are much more functional than those of percussion; they are developed by the patient himself, and of course cease with the termination of life. They are more complicated in their nature than the signs of percussion, and are less positive, because they may be modified by a greater number of circumstances: but when these are taken into the account, the deductions from auscultation are quite as conclusive.

The mode of practising auscultation is extremely simple; you may apply your ear directly to the chest, or you may interpose between it and the thorax of the patient a solid or flexible tube; hence auscultation is either immediate or mediate. As the sounds are produced by the patient, and not by your hand, as in percussion, you have thus far a very easy task; but you will find it of more difficulty when you come to the sounds themselves, and to their interpretation. Some, in themselves, are not easily learned; but others are difficult, only because they differ one from another by slight shades, and may therefore readily be confounded together.

For most purposes, immediate auscultation, or the direct application of the ear to the chest, is preferable to the use of a conducting tube. Those who are perfectly habituated to the exploration of the chest, prefer this method in the great majority of cases, on account of its greater rapidity and facility of application, for there is no previous preparation necessary, nor is there any difficulty in passing the ear rapidly over the chest. But in those portions of the thorax, where the space for the application of the ear is extremely limited, such as the clavicular regions and the axillæ, or above the mammæ in females, the stethoscope, as the conducting tube is called, will be found preferable. Besides these reasons of mere expediency, the sounds themselves are sometimes better characterized, or at least better limited in immediate auscultation; this is the case with the heart, and even with the lungs; for, as the instrument covers but a small space, and is perfectly isolated from the rest of the chest, the sound which is produced in the limited portion covered by its extremity, is alone conducted to the ear,—and that coming from the adjoining parts of the thorax is not heard, or at least is so feebly heard that it does not materially interfere with the result. When we apply the ear, we place the large surface of the head in contact with the chest, and as the bones of the chest and head are tolerably good conductors of sound, we hear the sounds of a larger portion of the lung than is desirable, and acquire less precise notions. But when you wish to examine rapidly a large portion of the chest, you will gain much time from this very circumstance, and take in at once the sounds from a large space, such as a whole lobe of the lung, or nearly so; and if you are really familiar with the sounds, they can be analysed and distinguished one from another, though heard at the same time, just as several instruments can be recognised in the same piece of music played by a complete orchestra. For ordinary purposes, therefore, immediate auscultation is much to be preferred.

When you use an instrument for conducting the sound from the chest to the ear, you will be obliged to take more precautions. This instrument is called a stethoscope, and is nothing more nor less than a tube of light wood, such as cedar; the extremity which is to be applied to the chest, is hollowed into the form of a cone, the apex of which terminates in the tube, and, of course, serves as an ear trumpet to conduct the sounds. The substance of the tube, although a comparatively good conductor of sound, is of much less service than the column of air; for an ordinary flexible ear-trumpet in which the sound is conducted exclusively by the column of air, is an excellent stethoscope. The diameter of the base of the cone should be from an inch to an inch and a quarter; if it be much larger, the sounds are confused, and the instrument loses its greatest advantage, that of concentrating the sound within a limited space; if too small, the sound is not loud enough. The essential point in the construction of a stethoscope is, that its cone should be deep and well hollowed out, at least an inch and a half or two inches deep, as is the case with several of the instruments which I now show you. The length of the tube may vary from four or five inches to a foot; six or seven inches will be found to be of a very suitable length for most purposes. The ear piece should be slightly convex or flat, or you may have a nipple-shaped projection, to insert into the ear; it should be of the same material as the rest of the tube, and not of ivory, as is often the case. Your ear will not be so near the chest as to expose you to the inconvenience of immediate auscultation, nor so far removed from it as greatly to diminish the intensity of the sound, for the sound becomes more gradually less and less loud in proportion as the ear is further removed from the part of the lung in which it is produced.

A flexible tube, that is an ordinary ear trumpet, about eighteen inches long, with the open end brought nearly to the form of the extremity of the stethoscope, is the best instrument for the examination of the sounds of the heart, as it does not conduct the impulse to the ear, hence the sounds alone are heard without the impulsion, which renders their analysis more difficult. Dr. Pennock of this city, who has devoted great attention to the diseases of the heart and their signs, was the first to introduce the flexible tube for this purpose, instead of the ordinary stethoscope.

I do not wish to detain you longer with the description of the mere instrument of hearing, which you may procure from any turner, but I must give you some cautions respecting the mode of application of the stethoscope. If you apply it directly upon the chest you must take great care that the end be placed flat upon the skin, without inclining to one or the other side, as the sounds are both modified and lessened by the admission of the air between the thorax and the tube. Indeed it is better not to place the instrument immediately upon the skin, but upon an under garment of muslin or flannel; this fills up the interstices between the tube and the surface, and prevents pain from too strong a pressure. The covering must be thin and not stiff, hence starched linen and silk are both improper, as they give rise to a rustling sound, and obscure the respiratory murmur.

The position of the patient for auscultation should be similar to that already directed for percussion, but the muscles and skin need not be drawn as tensely upon the ribs, for the pressure of the ear or the stethoscope against the chest will supply the effort performed by the muscles, and bring the parts as closely together as is desirable.

The signs derived from auscultation are divided into those of the respiration, of the voice, and cough, and lastly, of the heart. The signs of the respiration include both the modifications of the natural sounds produced by disease, and the rhonchi, or the new sounds, which are totally unlike those heard in the normal state. The latter class of signs are simple, and readily learned; the former are more important, and are produced by deeply seated alterations of the substance of the lung, producing a change in the density of its tissue. These signs are always attended with corresponding alterations in the percussion, and the resonance of the voice, which depend upon the same changes in the vesicular structure, and in the permeability of the bronchial tubes. They are thus learned, as it were, in connexion; and the signs of the respiration are strengthened or disproven by the corresponding changes in the voice and the percussion. Hence you will find it more easy to acquire them than it otherwise would be, for you may verify for yourselves at every step of your examination, and gradually acquire confidence in your powers of discrimination.

The morbid alterations of the respiration are well marked in extreme cases, but gradually pass into the characters of the healthy respiration; hence there is but one way of learning these signs. First to acquire the signs when strongly characterized, and then to proceed to the cases in which the modifications of respirations are but slight. In the diseased subject you will find that the strongly marked signs are very easily recognised; and many of you, who follow my practical demonstrations with sufficient attention, will naturally begin your study by the examination of patients who present these signs. Still, the facility for examining individuals in health is so much greater, that I should advise you all to familiarize and train your ear by the attentive study of those sounds presented by healthy individuals which approach most nearly to the signs of disease. And you will find that the characteristics of the radical sounds exist both in the healthy individual and in many diseased conditions.

These characters in healthy individuals are founded upon the peculiarities of the sounds in different parts of the chest dependent upon the differences in the tissue. The lungs consist of tubes conducting the air to vesicles in which the arterialization of the blood takes place. The sound of the air entering the vesicles is different from that caused by its passage through the tubes; and the former is therefore known as the vesicular sound, the latter as the tubal or blowing sound. The vesicular sound is often called a murmur, from its softness and diffusion over a large space, and cannot be produced unless the vesicles are healthy, or nearly so. If you keep up artificial respiration in an animal stunned by a blow on the head, or suddenly killed, and apply your stethoscope upon the exposed lung, the murmur is heard very distinctly during the inspiration, so that you have direct evidence that the sound is produced by the passage of the air into the vesicles; the vesicles, however, empty themselves in a noiseless manner, and the expiration is therefore nearly unheard. The tubal or blowing sound is quite different in its character; it is evidently produced by the passage of the air through tubes, and is heard very evidently both in the inspiration and expiration,—and is, in fact, much more distinct in the latter. The cause of this difference seems to be the different manner in which the air impinges upon the vesicles and tubes. During the inspiration the terminating point is of course the air vesicles,—and the air, if forced into them with tolerable rapidity, produces a sound; this is the same, whether the impelling force be the pressure of the atmosphere, upon the column of air in the bronchial tubes, when the parietes of the chest are elevated by muscular action, or to the force communicated by the bellows, when artificial respiration is carried on. This sound is in part owing to the vibration of the air, and in part to the noise produced by the dilating of the vesicles themselves. At least, the sudden dilatation of a partially collapsed vesicle is, in all probability, attended with sound, caused by the membranes; for, when the parietes of the vesicles are thickened, the sound probably becomes louder and more distinct. It is a point, however, which is difficult to decide, and one that is of little practical moment,—for, admitting either explanation, it is equally necessary that the vesicles should be clear, and that the air should pass freely into them from the adjoining tubes. The expiration produces a faint, vesicular sound; almost no sound in those portions of the lungs where the vesicular tissue is not traversed by bronchial tubes of a certain calibre. This probably depends upon the gradual manner in which the pressure upon the vesicles expels the air from them into the larger tubes through which it may readily pass towards the exterior: as the air is forced out from the vesicles very slowly, and of course not in a regular stream or current, they contract without sound. The vesicular murmur is compared to various sounds not very like to it; but it can be learned only in one way,—that is, by listening to those portions of the chest in which it exists in the greatest purity, especially towards the lower and lateral portions of the lungs. The murmur will be found to vary in intensity in different individuals: in some it is always feeble, and in others comparatively loud. It is loudest in those persons of a nervous temperament in whom the necessity for rapid respiration is greatest, than in stouter individuals. It is also louder in women and in children than in men and adults. The vesicular sound is indeed so much louder in children, that the term puerile respiration is used as synonymous with loud and full vesicular sound.

In most persons, the dilatation of the vesicles is obviously incomplete, except in forced inspirations, and in some is much more so than in others. This imperfect dilatation is rather more marked at the lower portions of the lungs than the upper, probably from the longer course and smaller size of the bronchial tubes, which require a more powerful effort to produce their full distension.

The tubal respiration is often called bronchial, from its production in the larger bronchial tubes,—or tracheal, from its development in the trachea,—the term tubal being thus confined to the most intense degree of this sound. In the healthy individual this may be heard in a very marked degree at the trachea, immediately above the sternum, and the air is then heard very easily as it passes through it, both in this inspiration and expiration. The sound is always blowing, and very different from the vesicular murmur; this character is best marked in the expiration. The cause of this difference will be very obvious to you if you attend to your own respiration; you will find then, if you breathe rapidly, that the expiratory sound, which is heard out of the chest, is much louder than the inspiratory, and is produced in the upper portion of the bronchial tree, and in the nasal fossæ, where the air passages are large, and the rapidity of motion of the air is greatest. It is for this reason, in the trachea the respiration is most decidedly tubal, or if you choose to use the term, tracheal. It gradually becomes less and less so as you approach more nearly to the parts of the lungs where the vesicular structure is most abundant, and contains tubes of the smallest calibre, and most removed from the surface. You may thus analyze the different sounds heard in various parts of the respiratory passages; you will find then the blowing sound only at the trachea, and the vesicular only at the lower parts of the chest,—while at the roots of the lungs there is a mixture of the two varieties of sound, and the vesicular is combined with the blowing sound. Passing from the root of the lung you will find a gradual diminution in the loudness of the bronchial sound,—but it is still heard as far as the summit, and much more distinctly on the right side than on the left. The difference in the two sides arises from the anatomical structure; for the tubes leading to the upper part of the right lung are shorter and larger than those going to the left, on which side the large bronchus passes under the aorta, and is therefore much longer and more tortuous than upon the right. The larger but shorter tubes of course approach much more nearly than the longer and smaller ones to the physical condition of the trachea, in which the air circulates with such freedom as to give rise to the loudest double blowing sound. The louder blowing sound exists on the right side, both at the anterior and posterior part; hence a given amount of induration of structure, which may tend to increase the loudness of this sign, will be much more perceptible on the right side than on the left,—while, on the other hand, in the state of health, a perfectly natural peculiarity may be mistaken for disease. The blowing sound, if it be heard only on the right side, must be well characterized to become a sign of disease, and is not of much value unless combined with other corroborative evidence.

This difference of respiratory sound on the two sides of the chest dependent upon the different structure of the lungs, was not pointed out previously to some researches which I undertook upon the subject, at the Children's Hospital of Paris, about eight years ago. My attention was called to the subject by the observation made by my lamented friend Dr. Jackson of Boston, who laid great stress upon the characters of the expiration observable in commencing phthisis, and other diseases attended with consolidation of the lung. His remarks upon the early development of the blowing expiration in commencing phthisis, were perfectly well founded; but at the commencement of his researches he was sometimes led into error from not making due allowance for the difference of the two sides dependent upon peculiarities of conformation.

In the study of the respiration you have a plain course to follow: examine as often as you can the region of the trachea, and then the lower and vesicular portion of the lungs, and thus fix in your minds the difference between the two leading varieties of the respiration, or the tubal and the vesicular. Some of you find this study a matter of some difficulty, while others can seize the distinctive characters at their first effort. You must not be in doubt as to the cause of this difficulty when it exists; it arises in part from a less acuteness of hearing, but much more from a defect of attention, which you may readily supply by your own efforts; and, rely upon it, you know nothing of auscultation until you have mastered this subject. After the best marked sounds are learned, you may proceed to those parts of the chest in which you will hear the two varieties of the respiration at the same time; you then analyze their peculiarities, and may ask yourselves at each moment whether you have a clear idea of both sounds, as they are heard together. The same process should be repeated in different individuals of various ages, sex, and conformation; and you will find that although they present numerous shades of difference, the radical features are the same, and must always be the same, for they depend on known principles of acoustics.

In connexion with this part of your studies, you may properly enough accustom yourselves to the shades of difference offered by the parts of the lung, where other viscera, such as the heart and liver, occupy a portion of the space beneath the ear, and you may in this way learn the abrupt manner in which the respiration generally ceases at the level of the lung. During your examination you should direct the patient to breathe with different degrees of rapidity, sometimes quite naturally, and at others much more quickly, so as to force the air into the vesicles. In the examination of diseased individuals we follow nearly the same order,—and, after placing our ear for a moment upon the chest of the patient while breathing in a quiet and regular manner, we usually direct him to make a forced inspiration, which clears out the mucus in the bronchial tubes, and supplies a full proportion of air to each vesicle. In cases of disease of an acute character obstructing a portion of the lung, there is no necessity for directing the patients to breathe rapidly, as the obstruction in the diseased part of the lung causes the respiration in the rest of the pulmonary tissue to be much exaggerated or puerile.

After the radical characters of tracheal or bronchial respiration, which differ merely by a shade, of the vesicular respiration, and of the rude respiration, which is intermediate to the two leading varieties are learned, you may proceed to the study of the morbid alterations of the respiratory sounds. These are classed according to their greater or less accordance with the natural characters of the respiration. I will begin with the most strongly marked. This is the bronchial respiration, and its varieties, which include the cavernous and amphoric respiration. The bronchial respiration, as it occurs in a diseased lung, is essentially the same with the tracheal respiration of the healthy chest. The bronchial respiration is developed by causes which harden the parenchyma of the lungs, and destroy the vesicular texture: these are the infiltration of the tissue of the lungs with blood and plastic lymph in pneumonia, the compression of the lung by pleuritic effusions, and the deposits of various anomalous productions, such as tubercle and cancer, in the tissue of the lungs. If the induration be seated around the larger bronchial tubes, the bronchial respiration is much louder than in the portions of the lung where the tissue is chiefly composed of vesicles, for the essential cause of this sound is the passage of the air through the tubes; the induration of the substance of the lung is merely a favourable circumstance which developes the sound where it is not generally heard, or increases it in those parts of the lung in which it exists naturally. The bronchial respiration is produced then partly by the obliteration of the vesicles, and partly by the closure of the smaller tubes. Hence, the air in passing through tubes of a certain size is suddenly interrupted and repelled from their sides, because their terminating branches are closed. This repulsion produces sound, and increases the blowing respiration, which is heard most loudly when the air, instead of diffusing itself throughout the vesicular tissue, is, on the contrary, forced through the larger bronchi, which are converted into closed cylinders, from the occlusion of their branches by the progress of the disease.

The bronchial respiration is often accounted for in the following way: The passage of the air in the tubes is, under ordinary circumstances, not attended with sound; as the surrounding tissue is a bad conducting medium, and deadens the sound. When this tissue is rendered more solid, the sound already produced in the tubes becomes audible, and is conducted to the ear. This explanation is valid only to a certain extent; the sound heard exists only in a slight degree in the natural state, for a tube through which the air is constantly and equally drawn during the respiration, gives rise to a very faint sound, but if the tubes passing into it be cut off, the passage of the air is at once hurried by its friction against the parietes of the bronchus, giving rise to the usual bronchial sound. In disease, therefore, the blowing sound is very often much louder in those portions of the lung where it does not exist naturally, than over the trachea or the larger tubes, which are almost immediately beneath the ear; and this extreme loudness depends upon the circumstance to which I have already alluded,—that is, the sudden reflection of the column of air from the interrupted tube.

The large size of the tubes is, however, as I have already stated, a circumstance highly favourable to the development of bronchial respiration; and if the tubes be superficial, the influence of size becomes more obvious. If the tubes be enlarged, while the parenchyma remains healthy, the respiration is bronchial, but to a less degree than if the tissue be hardened, and the tubes retain their usual calibre; for the induration is a more efficient cause of bronchial respiration than simple enlargement of the tubes.

The bronchial respiration is not perfect except when the induration of the pulmonary tissue is complete; this takes place in a few cases of phthisis, and in pleurisy with large deposit of lymph, but is much more frequent in pneumonia than in any other disease, for in none other is the hardening of the tissue so perfect: this sign is therefore one of the best indications of the second stage of inflammation. In dilatation of the tubes the respiration becomes very bronchial when the surrounding tissue is indurated, that is, when complicated with pneumonia.

The bronchial respiration, then, is produced by the passage of the air through tubes of the middle and larger size in an indurated lung, and also by the enlargement of these tubes. The cavernous respiration is another variety of sound which is closely analogous to the bronchial respiration, and depends upon the passage of the air into a cavity communicating with the bronchi. For physical purposes this cavity may be considered as a mere dilatation of the bronchus with which it communicates; but as the termination of the tubes themselves is never so abrupt as the morbid cavity, the air in the bronchial respiration proper is gradually diffused through the tissue, and is slowly lost,—but in the cavity it is abruptly reflected from the walls of the excavation, and therefore seems to be more circumscribed, and comes from a limited point. This diffusion of sound in the one case, and concentration in the other, constitute the difference between these variations, and they therefore run into each other by insensible shades. As the line of distinction is an arbitrary one, it is sometimes impossible to discriminate between them, but it is not generally a matter of much practical moment, for the signs of a cavity generally become more and more distinct in proportion to the duration of the disease, and the doubtful usually become clear in a short time.

The amphoric respiration is a modification of the same sound, but is more unlike the bronchial respiration. It is produced by the passage of the air into a large cavity with firm walls. If the communication between the cavity and the bronchi be free, the expiration is also loud, and the sign differs from the cavernous respiration in one respect only—it is fuller and more musical, somewhat similar to the sound caused by blowing into a glass or metallic vessel. Both the inspiration and expectoration are blowing, and there is no trace of the vesicular murmur. If the communication with the bronchi be interrupted, or too small to allow of the free passage of the air, the inspiration alone is distinctly heard, as the air passes out of the cavity too slowly to produce much sound. The common cause of amphoric respiration is a large tuberculous cavity near the surface, and surrounded by indurated lung. It may also depend upon perforation of the pleura; in which case the amphoric tone is extremely well marked, as the cavity is much larger than one formed in the lungs, and its walls are large and elastic. If the amphoric respiration depend upon a gangrenous cavity, it is generally less marked than in tuberculous excavations, as the surrounding tissue is usually soft, and is therefore a bad conductor of sound.

We now return to the bronchial respiration as our standard of comparison, and pass from it to the vesicular murmur, reversing the order we have just followed. The varieties of the respiration intermediate between the vesicular murmur and the true bronchial respiration, are very numerous, but they are properly enough classed under the general designation of rude, or rough respiration, which is applied to those varieties in which the vesicular murmur is still retained, but the blowing sound is at the same time more developed than is natural in the part of the lungs where it is heard. It may be attended with a feeble or an increased loudness of the vesicular murmur. When this is more feeble, the obstruction to the air occurs about the smaller tubes, and gradually compresses them; when loud, the morbid deposit is situated rather in the course of the larger tubes than at their terminating branches, which still receive their full supply of air while the respiration becomes blowing from the increased conducting power of the hardened tissue. The rude respiration is one of the most interesting varieties of the respiratory sound, for it occurs in those varieties in which the lesion is not yet much advanced, and a portion of the pulmonary tissue remains permeable to the air; hence it is a sign of the earlier stages of phthisis, of the commencement of pneumonia and of pleurisy. It is a sign which you will learn with some difficulty, because both the primitive varieties of the respiration are present, and they can only be separated by a careful analysis.

From the rude respiration we naturally return to the vesicular murmur; which may be exaggerated, or enfeebled, but still retain its essential characters. The exaggerated or puerile respiration, generally depends upon disease in other portions of the lungs than those in which it is heard. The healthy portions then perform double duty, and arterialize more than their proper share of blood. From the occurrence of puerile respiration in a part of the lung of a patient who labours under dyspnœa, we can very often determine that some obstruction must exist in other parts of the lungs; and from the knowledge of the acute and chronic diseases which generally give rise to this obstruction, we can with tolerable certainty discover the nature of the lesion. The respiration is rendered feeble in disease, either by the compression of the vesicles from effusion upon the exterior of the lung, or the development of solid matter in the parenchyma, or lastly, from obstruction of the smaller tubes.

There are some other varieties of the respiration, which it were difficult to bring within a systematic description; they should be learned after the leading varieties have been first studied. They generally arise from slight changes in the condition of the vesicles or smaller tubes, and sometimes from the mode in which the respiration is performed, but rarely depend upon important organic changes in the lung. They may be reduced to the following: 1st, the incomplete or interrupted respiration; in this variety the inspiratory sound seems to be arrested before the air passes completely into the vesicles; it arises from two causes,—a nervous spasm, and a partial thickening or congestion of the smaller tubes. It is a peculiarity which is often observed when we examine for the first time a nervous, sensitive patient, who is alarmed by the exploration of the chest; and it is sometimes met with in the infiltrated or congested state of the lungs which attends the forming stage of tuberculous disease, as well as certain varieties of bronchitis. 2d. The rustling sound of the respiration is one of the characteristics of emphysema, in which the vesicles dilate and contract with difficulty, and seem to produce sound rather from the rustling of the membrane, than the air which impinges against it.—There are other and slighter deviations from the natural tone of the respiratory murmur; but, although they are very obvious to an experienced eye, yet they are neither sufficiently permanent nor well marked to be reduced into a systematic classification.

The varieties of the respiratory sound correspond with varieties in the resonance of the voice, which often are nearly as well characterized; still, the natural tone of the voice has so much influence upon its aptness for vibration, that the signs are not always as perfectly distinctive as those of the respiration. In the ordinary act of speaking the voice vibrates throughout the chest; and if the hand be placed upon its parietes, a slight tremour is very perceptible; if you apply your ear to it, you will hear a thrilling, but distant and confused sound. This sound becomes louder, and is brought nearer to the ear, if you listen near the summit of the lungs, especially on the right side, or at their root; and if you then place the stethoscope upon the trachea, you find the resonance loud, and the words pronounced nearly as distinctly as they are by the mouth. In fact, the voice is conducted by the column of air, and then articulated words seem to enter the ear from the trachea. This distinct and loud resonance at the trachea is pectoriloquy; and it is in this situation very perfect, especially if the voice of the individual be naturally clear, and rather shrill. At the sternum, and at the root of the lungs between the scapulæ, the resonance is less perfect, and the voice seems to enter the ear less completely than in pectoriloquy; it is therefore not quite so well characterized a sign, and is called from its position, bronchophony. In the rest of the lung the resonance of the voice is gradually less and less as you pass from the bronchi to the vesicular structure, where you hear nothing but a faint vibration.

There is, therefore, a uniform relation between the voice and the respiration, the resonance of the voice being greatest when the blowing sound of respiration is most intense. In disease the same proportion exists; a cavity gives rise to cavernous respiration in breathing, and to pectoriloquy in speaking,—and a consolidated lung, especially around the large bronchi, produces bronchophony and bronchial respiration. The same relation exists between a mere loud resonance of the voice and rude respiration, and between the ordinary vesicular murmur and a slight thrilling vibration of the voice. In cases in which the murmur is enfeebled, the resonance of the voice is less; but sometimes there is a low, purring sound, communicated to the ear as well as the hand, which is analogous to the rustling sound of emphysema, and depends upon the same causes. The blowing respiration may continue very loud when the resonance of the voice has become quite feeble, for an accumulation of mucus may be forced aside by a full inspiration, but cannot be thrown out of the way by the act of speaking, and therefore obstructs the vibration of the column of air: in these cases it is not, however, totally destroyed, for the sound is conducted by the hardened lung from the neighbouring tubes.

When a cavity in the lungs is very large, there is, of course, amphoric respiration at the same time. You will then find amphoric resonance of the voice, which often scarcely differs from pectoriloquy: that is, if the cavity be not much larger than a hen's egg, and its walls remain firm. But if the cavity increase much beyond this size, the resonance of the voice is extremely metallic, or has a clear ringing sound, which, like the respiration, is very similar to that produced by speaking in a glass bottle without quite closing its mouth.

But when the large cavity is situated in a soft permeable portion of the lung, the amphoric respiration may be obscure, like the resonance of the voice under the same circumstances.

The bronchial respiration which results from pleuritic effusions, differs so slightly from the other varieties, that it is usually not separated from them, but the resonance of the voice which takes place under the same circumstances, is very different. Its vibration is very great, and is so peculiar that the sound is called egophony, from the bleating tone of the voice, somewhat similar to that of a goat or sheep. This is not an invariable result of pleuritic effusions, but it is produced in all cases in which the effusion is sufficient to compress the lung without entirely flattening it out. If the quantity of liquid happens to be very great, but the lung stiff and more solid than usual from previous inflammation of its substance, its egophony continues longer than it otherwise would do, and rarely ceases during the course of the disease.

The signs of the voice are learned by the same process as those of respiration. After having acquired a good general idea of the characters of the respiration, you should examine them in connection with the signs of the voice, confirming or disproving one by the other, and then practising percussion, which will throw additional light upon the subject. You need not, of course, restrict yourselves to the healthy subject, but you may also study those cases of diseased lungs in which the diagnosis is comparatively easy from the functional signs, such as examples of decided phthisis and pneumonia, and then search for cavernous and bronchial respiration, with the connected signs of the voice and percussion.

The cavernous resonance of the voice in pectoriloquy was the first physical sign discovered by Laennec. He happened to place some paper rolled up into the form of a cylinder upon the chest of a patient, in order to feel the pulsations of the heart, when he was surprised to find that, during the act of speaking, the voice of the patient seemed to enter his ear. He examined immediately the chest of a large number of patients in the same way, and detected the same phenomena in a great number who were evidently labouring under advanced phthisis; the cause of this was afterwards found to be cavities in the lung communicating with the bronchial tubes. Pectoriloquy was divided by him into three varieties, the perfect, the imperfect, and the doubtful; in the perfect, the voice seemed to pass through the stethoscope (which Laennec always used) to the ear, in the second to enter the tube, and in the third the resonance was quite confused. These distinctions are of little value and rather tend to confuse your ideas.

The following table will give you the relation between the voice and the respiration.

| | |
|---|---|
| Amphoric Respiration, | Amphoric Resonance of Voice, |
| Cavernous Respiration, | Pectoriloquy, |
| Bronchial Respiration, | Bronchophony, |
| Rude Respiration, | Strong Resonance of Voice, |
| Vesicular Respiration, | Slight Thrilling of Voice. |

